# Motor priming in virtual reality can augment motor-imagery training efficacy in restorative brain-computer interaction: a within-subject analysis

**DOI:** 10.1186/s12984-016-0173-2

**Published:** 2016-08-09

**Authors:** Athanasios Vourvopoulos, Sergi Bermúdez i Badia

**Affiliations:** 1 Faculdade das Ciências Exatas e da Engenharia, Universidade da Madeira, Campus Universitário da Penteada, 9020-105 Funchal, Portugal; 2 Madeira Interactive Technologies Institute, Polo Científico e Tecnológico da Madeira, Caminho da Penteada, 9020-105 Funchal, Portugal

**Keywords:** Stroke rehabilitation, Brain-computer interfaces, Virtual reality, Motor priming, Motor imagery, Neurofeedback, EEG

## Abstract

**Background:**

The use of Brain–Computer Interface (BCI) technology in neurorehabilitation provides new strategies to overcome stroke-related motor limitations. Recent studies demonstrated the brain's capacity for functional and structural plasticity through BCI. However, it is not fully clear how we can take full advantage of the neurobiological mechanisms underlying recovery and how to maximize restoration through BCI. In this study we investigate the role of multimodal virtual reality (VR) simulations and motor priming (MP) in an upper limb motor-imagery BCI task in order to maximize the engagement of sensory-motor networks in a broad range of patients who can benefit from virtual rehabilitation training.

**Methods:**

In order to investigate how different BCI paradigms impact brain activation, we designed 3 experimental conditions in a within-subject design, including an immersive Multimodal Virtual Reality with Motor Priming (VRMP) condition where users had to perform motor-execution before BCI training, an immersive Multimodal VR condition, and a control condition with standard 2D feedback. Further, these were also compared to overt motor-execution. Finally, a set of questionnaires were used to gather subjective data on Workload, Kinesthetic Imagery and Presence.

**Results:**

Our findings show increased capacity to modulate and enhance brain activity patterns in all extracted EEG rhythms matching more closely those present during motor-execution and also a strong relationship between electrophysiological data and subjective experience.

**Conclusions:**

Our data suggest that both VR and particularly MP can enhance the activation of brain patterns present during overt motor-execution. Further, we show changes in the interhemispheric EEG balance, which might play an important role in the promotion of neural activation and neuroplastic changes in stroke patients in a motor-imagery neurofeedback paradigm. In addition, electrophysiological correlates of psychophysiological responses provide us with valuable information about the motor and affective state of the user that has the potential to be used to predict MI-BCI training outcome based on user’s profile. Finally, we propose a BCI paradigm in VR, which gives the possibility of motor priming for patients with low level of motor control.

## Background

Brain-Computer Interfaces (BCIs) are communication systems capable of establishing an alternative pathway between user’s brain activity and a computer system. The most common signal acquisition technology in BCI is the non-invasive electroencephalography (EEG) [1]. The EEG activity is distinguished by different wave patterns in the frequency domain called EEG bands or rhythms. These EEG rhythms are divided into different ranges including Alpha (8 Hz - 12 Hz), Beta (12 Hz - 30 Hz), Theta (4 Hz - 7 Hz), and Gamma (25 Hz - 90 Hz) and each rhythm had been found to be related with sensorimotor and/or cognitive states [2, 3]. Rhythms in the Alpha and Beta frequency bands are functionally related to major sensorimotor systems [4] which are activated primarily through motor preparation or execution [5]. Alpha and Theta oscillations are known to reflect cognitive and memory performance [6, 7], and Theta was shown by early EEG studies to be closely related with problem solving, perceptual processing and learning [8]. Finally, Gamma rhythm has been shown to be modulated during volitionally meditation, consciousness, and sense of self [9]. In addition, decreased levels of Gamma is observed in children with ADHD [10], in Alzheimer's Disease (AD), and also in epileptic patients [11]. Overall, EEG signals offer low spatial resolution measures of neural activity that occurs in the cortical area of the brain. Translating cognitive states or motor intentions from different rhythms is a complex process and is impossible to associate a single frequency range or cortical location to a brain function.

For BCIs, this oscillatory brain activity -recorded through EEG- is currently used for the interfacing between humans and computers. This communication can be triggered by an exogenous stimulus through visual, auditory or sensory feedback, like Steady State Visual Evoked Potentials (SSVEP) and P300. SSVEP is caused by visual stimulation of flashing lights and occur at the primary visual cortex of the brain [12]. Instead, P300 responses are generated by measuring the brain evoked responses 300 ms after stimulus onset (hence the name) [13]. In contrast to exogenous sources, motor-imagery (MI) BCI is of endogenous origin and makes use of the visuo-motor imagination (imagination of upper and/or lower limb movement). MI has been shown to share common control mechanisms and neural substrates of actual movement both in action execution and action observation [14], providing a unique opportunity to study neural control of movement in either healthy people or patients [15, 16]. Therefore, MI has been widely used as the main BCI paradigm in research [17] for individuals with high degree of motor disability or locked-in syndrome [18, 19]. To date, MI is proven useful in a wide area of applications ranging from accessibility tools for disabled users with paralysis or severe neuromuscular disorders [1], for restoration of active movement [20], to human-computer interaction research [21], virtual reality and video games [22].

In stroke rehabilitation, BCIs have been mostly used with two different strategies. The first one is the “assistive”, which aims at bypassing non-functional corticospinal pathways for controlling robotic prosthetics [23]. The second is the “restorative”, which aims at mobilizing neuroplastic changes in order to achieve the reorganization of motor networks to attain functional motor recovery [24]. For the latter case, MI-BCI training has been the most widely used BCI paradigm [17]. Results from previous studies have proven mental practice of action to be useful in MI-BCI training [25]. MI training is leading to the activation of overlapping brain areas with actual movement, and because sensory and motor cortices can dynamically reorganize through neuroplasticity [26, 27], MI constitutes an important component for motor learning and recovery. Moreover, research about the mirror neuron system (MNS) have shown that action observation, motor imagery, and imitation share the same basic motor circuit as action execution and thus provide an additional or alternative source of motor training that may be useful to promote recovery from stroke [28]. In addition, it has been found that the spatial distribution of local neuronal population activity during MI mimics the spatial distribution of activity during actual motor movements [29]. Beneficial effects of MI in motor control have been shown [19], and new paradigms have been proposed to maximize the recruitment of motor networks [30]. In stroke rehabilitation, the combination of BCIs with virtual environments has gained popularity, and it has been shown very useful to train functional upper limb pointing movements [31, 32]. Unfortunately, MI-BCI studies for stroke rehabilitation are very different in terms of (a) experimental design and (b) research protocols. So far in MI training, the use of abstract feedback in the form of unidirectional arrows as the main visual feedback mechanism is the most widely used [33]. Although there is no direct evidence that different feedback designs, i.e. realistic grasping with a hand vs. extending arrows, imply differences in performance in MI [15], previous studies have shown that the type of feedback can have different effects based on the learner [34]. For instance, emotional feedback (in the form of smiley faces) has shown positive results in MI performance [35]. Other researchers have studied the effect of alternative feedback modalities on a BCI task, such as haptic and auditory feedback, with inconclusive results [36, 37]. Interestingly, it has been shown that the combination of audio and visual feedback decreases BCI performance, whereas the combination of haptic and visual feedback increases the performance [38, 39]. In another experiment, displaying real-time cortical activity as neurofeedback was shown to significantly increase MI performance [40]. Furthermore, videogames and Virtual Reality (VR) feedback has also produced positive results, offering a more compelling experience to the user through 3D environments [22, 41]. The fusion of BCI and VR (BCI-VR) allows a wide range of experiences where participants can control various aspects of their environment -either in an explicit or implicit manner-, by using mental effort alone. This direct brain-to-VR communication can be used to induce illusions mostly relying on the sensorimotor contingencies between perception and action [42]. Friedman et al., in a study from 2007, included three different BCI setups: (i) abstract feedback, (ii) head-mounted display (HMD), and (iii) the CAVE-like system. They did not find any consistent performance trend related to the type of interface, but the event-related synchronization (ERS) was stronger in the CAVE setup [43].

Despite the increased attention that BCI technology has had with the launch of low-cost commercial EEG devices in the last few years, BCI technology is hardly used outside laboratory environments [41]. Unfortunately, BCIs are not yet as accurate as other types of interfaces [44], and users require a training period up to several months to achieve accuracies of 65 %–80 % using cortical potentials [1]. Although accuracy varies among the different BCI paradigms, most are not 100 % accurate, they require extensive training, and have low information transfer rates and long response delays [45]. For instance, MI-BCI requires long training trials and settings are subject specific. As consequence, long and repetitive training sessions can result in user fatigue and declining performance over time. In addition, prolonged training is problematic in generating the EEG oscillatory rhythms modulated during MI, such as Mu and Beta rhythms [3]. New findings in MI experimentation have shown that increased vividness of imagery is strongly associated with the neural activity in motor related areas [46] and that the kinesthetic imagination of movement is preferable over just visual imagination, resulting in increased MI-BCI performance [47]. Unfortunately, there is a limited understanding on how these factors affect the activity patterns of motor related areas. Recent studies have shown that physical activity prior to a MI task (motor priming) facilitates the engagement of motor networks on the subsequent MI task [48]. It has been shown that during feedback presentation EEG synchronization patterns increase hemispheric asymmetry compared to control sessions without feedback [49]. In addition, hemispheric asymmetry is related with increased performance of fine motor tasks, and specifically left hemisphere changes are related to motor learning [50]. However, different studies had different experimental setups and it is not clear how we can improve the design of a MI-BCI paradigm. Moreover, there is a lack of systematic studies dedicated to the actual aspects of the experimental (training) task, focusing mostly on the technical aspects of the system. Therefore, in the area of neurorehabilitation there is an urgent need to identify the key elements for a successful MI-BCI training using specific criteria for motor rehabilitation for including patients with severe hemiparesis. This leads to questions such as, (1) How can we include patients with low level of motor control, (2) how can we maximize both performance and sensorimotor activation, and (3) how can we promote adherence to MI-BCI training?

In order to overcome some of the limitations of current BCI systems, we performed a study based on a novel prototype that makes use of multimodal feedback, in an immersive VR environment delivered through a state-of-the-art Head Mounted Display (HMD), integrated in a MI-BCI motor training task (left | right hand imagery) [51]. To achieve maximum engagement of sensory-motor networks in a MI-BCI motor rehabilitation task, we assessed the role of motor priming and multimodal VR feedback compared to a control condition. In this study we included naïve subjects, with no previous exposure in BCI, in order to have a first-time user experience (FTUE).

Based on the above analysis of the literature we expect that:

1. Through an immersive multimodal VR environment and motor priming, we can maximize the engagement of sensory-motor networks important in neurorehabilitation, due to the enhanced modulation of the same cortical areas that are activated during actual motor preparation and execution.
2. We can quantify the relationship between users’ electrophysiological data and psychophysiological responses, important for identifying which patient profile can benefit the most from an immersive BCI-VR setup for MI training.

## Methods

### Experimental design

**Fig. 1:**
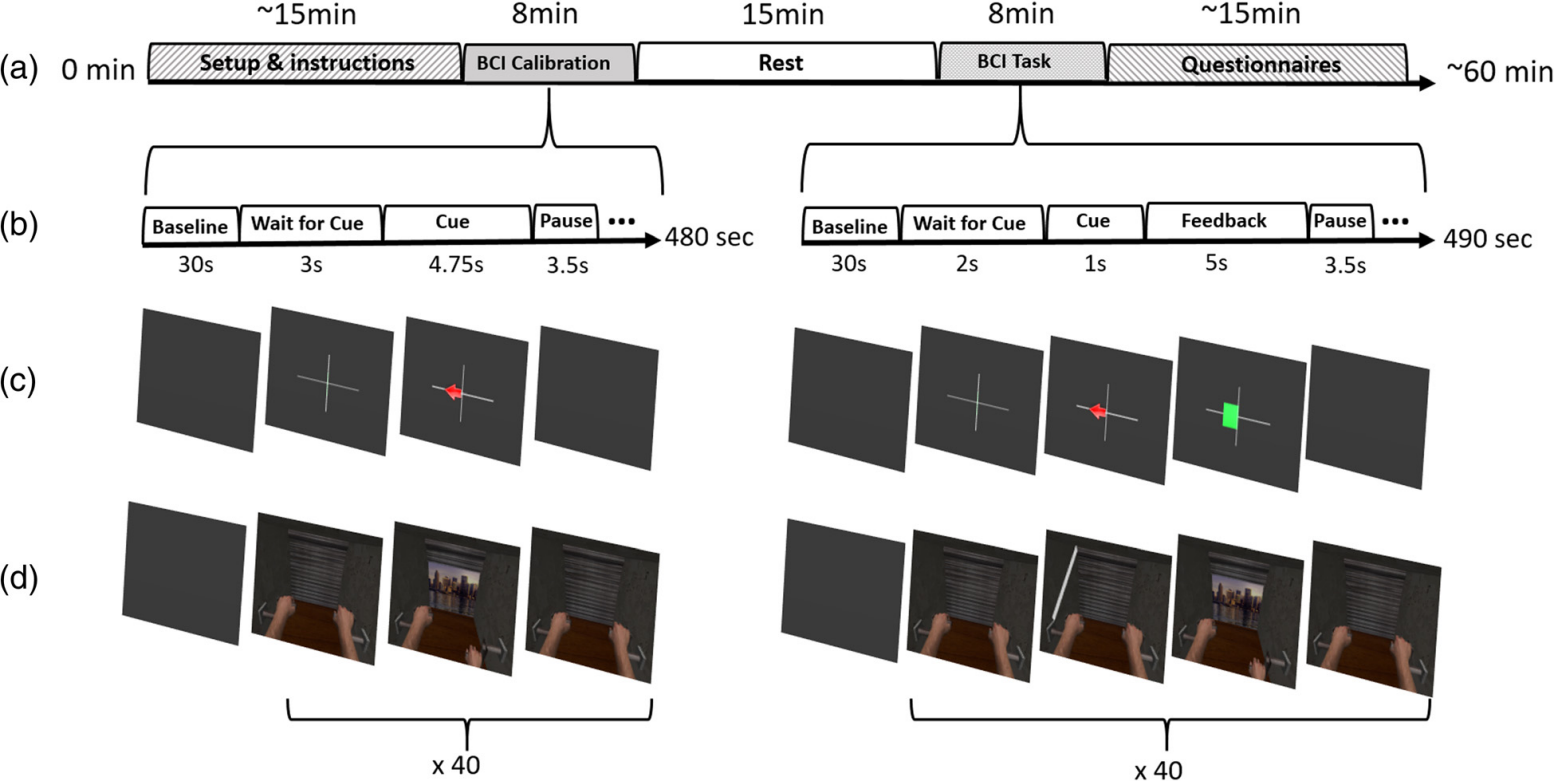
Experimental Setup overview. (**a**) Experiment timeline, starting with a 15 min briefing and setup, followed by 8 min of BCI calibration, 15 min of rest, 8 min of online task performance, and questionnaires. (**b**) BCI calibration and MI task blocks. Starting with baseline measurement, the user waits for a cue followed by a pause (repeated 40 times, 20 per class). (**c**) Stages for the Control condition with the standard arrows-and-bars feedback. (**d**) Stages for the VR training feedback, replacing the directional arrows with virtual hands performing a task in a 3D immersive environment

In this experiment we used a within-subject design. The protocol consisted of 3 BCI conditions to which users were exposed in a randomized order, and their EEG activation patterns were then also compared to the activity during overt motor-execution. Each participant performed one condition per day, completing all conditions in 3 days. Each condition included 5 main blocks (Fig. 1): (1) 10–15 min of equipment setup and instructions; (2) subjects were then exposed to an 8 min MI-BCI calibration block followed by (3) a 15 min pause; (4) a MI-BCI task of 8 min; and (5) subjects answered a set of self-report questionnaires. In total, each condition lasted approximately 60–70 min with 16 min of BCI exposure. During all blocks in all conditions, EEG data were logged synchronously and time-stamped including the different stimulation codes [Start of trial, End of trial, Left, Right, Feedback, Cross on screen] for offline analysis.

### Experimental conditions

In our design of the BCI setup, we incorporated properties that are recommended as a good instructional design in BCI training [41]. In all conditions we presented the user only with the correct classified action for enhancing the feeling of competence, we provided a clear and meaningful task through the virtual task paradigm, the task was self-explanatory, simplified and intuitive, with progress of achievement, challenging but achievable, and finally in an engaging 3D virtual environment. All 3 BCI conditions were designed based on the Graz-training paradigm [52]. The control condition incorporated the Graz-training with abstract bars-and-arrows feedback, and for the VR version we used ambient and event sounds and a virtual representation of two hands performing the motor action.

Three experimental conditions were designed with different feedback and priming mechanisms: multimodal VR with motor priming, multimodal VR, and standard MI. For all conditions, a total of 10 repetitions (of approximately 4 s duration, followed by a 2 s pause) of motor-execution/mental simulation for each hand were performed and presented always through a HMD.

### Multimodal Virtual Reality with Motor Priming (VRMP)

**Fig. 2:**
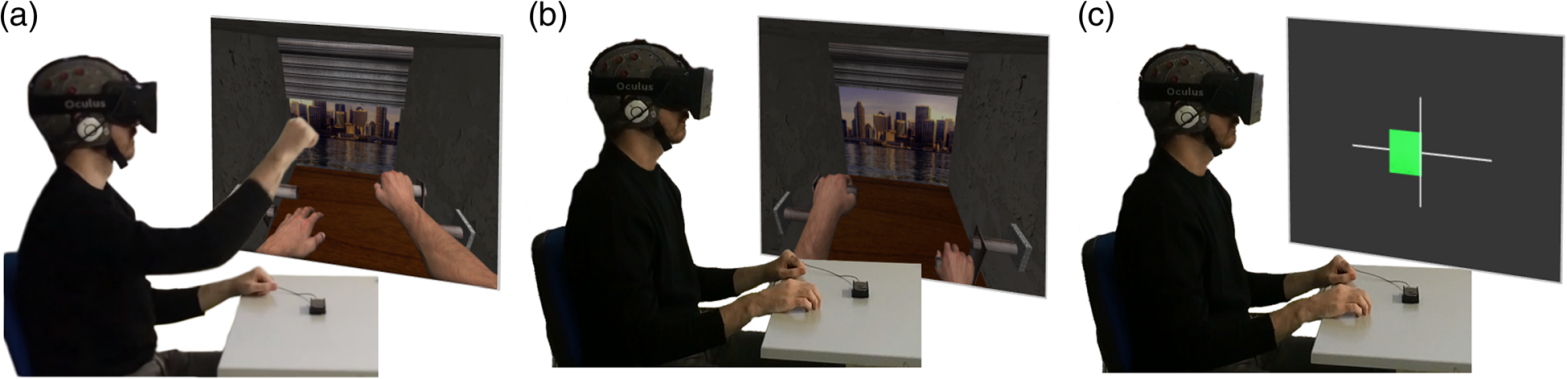
MI-BCI training conditions. (**a**) VRMP: the user has to perform motor priming by mapping his/her hand movements into the virtual environment. (**b**) VR: the user has to perform training through simultaneous motor action observation and MI, before moving to the MI task were he/she has to control the virtual hands through MI. (**c**) Control: MI training with standard feedback through arrows-and-bars

In this condition, users were asked to carry out a motor-execution task for 8 min using an immersive virtual reality environment before performing the MI-BCI calibration block. For this, we combined the HMD with a natural user interface that tracked hand and finger movements to enable a natural interaction of the participants with the virtual environment, by mapping the movement of their own hands to VR with an update frequency of the visual feedback at 30Hz (Fig. 2a). The motor-execution task, a “virtual garage”, involved the rotation of a virtual lever through circular movements for opening a large garage door. The virtual environment included spatial sounds related with the movement of the door and the lever. The sounds generated by the chain mechanism and other mechanical sounds, were activated through the rotation of a handle that controls the opening of a virtual garage door. Before each repetition, the user was informed of which hand should be used to open the garage door. This stage will be further referred as motor priming (MP) block. Subsequently, a MI-BCI calibration block took place to determine the best MI classifier parameters based on the same VR task and feedback as used during MP. In this block, the user had to imagine the same movement performed previously in the MP block. Finally, the same virtual environment was used for a MI-BCI online block, in which the user could directly control the virtual arms through the BCI interface using MI.

### Multimodal Virtual Reality (VR)

In this condition, users were asked to only carry out the MI-BCI calibration block and the online MI-BCI task block as in the previous condition, but without the prior MP (Fig. 2b).

### Control - standard motor imagery

In this condition, a standard MI-BCI paradigm was used, providing a control condition for the other conditions to be compared with. Hence, this condition followed the same protocol as the VR condition, but instead of the VR component only simple bar-and arrow-elements without sounds (the so-called Graz visualization) were used as feedback mechanisms (Fig. 2c). Yet, the MI task consisted in the motor imagery of the same upper-limb movements as described in conditions VRMP and VR and was presented through the same HMD.

### Experimental setup

The experimental setup was composed by a desktop computer (OS: Windows 8.1, CPU: Intel® Core™ i5-4440 at 3.3 GHz, RAM: 8GB DDR3 1600MHZ, Graphics: Nvidia GT 630 1GB GDDR3), running the 3 different MI-BCI training conditions described above. All visual and auditory feedback was developed with the Unity 3D game engine (Unity Technologies, San Francisco, USA). For hand and finger tracking during the MP block, the Leap Motion controller (Leap Motion, Inc., San Francisco, California, United States) was used to map hand and finger movements to the virtual counterparts. A stereo headset for spatial sound was used in VR and VRMP conditions. The Oculus Rift DK1 HMD (Oculus VR, Irvine, California, United States) was used for all conditions, regardless of the feedback modality.

The BCI set up consisted of 8 active electrodes equipped with a low-noise biosignal amplifier and a 16-bit A/D converter at 256 Hz (g.MOBIlab biosignal amplifier, gtec, Graz, Austria). The spatial distribution of the electrodes followed the 10–20 system configuration with the following electrodes over the sensory-motor areas: FC3, FC4, C3, C4, C5, C6, CP3, and CP4. The signal amplifier was connected via bluetooth to a laptop computer (CPU: Intel® Core™ i3-3217U at 1.80 GHz, RAM: 8GB DDR3 1600MHZ, Graphics: Intel® HD Graphics 4000) for the EEG signal acquisition and processing through the OpenVibe platform [53]. For all conditions, a Common Spatial Patterns (CSP) filter was used for feature extraction, based on the mutual diagonalization of each covariance matrix for each class to be discriminated [54]. CSP has been shown to deliver better performance in MI experiments [55]. In addition, Linear Discriminant Analysis (LDA) was used for the classification of the two classes (left | right hand imagery) from the feature vector. LDA reduces the dimensionality of the data and establishes a surface decision in the feature space which separates data into two groups, each one related to one class [56]. Finally, the classified data were transmitted to the RehabNet Control Panel (RehabNetCP) [57] through the VRPN protocol [58] to control the virtual environment. The RehabNet Control Panel is a free tool that acts as a device router to bridge a multiple interfaces with virtual environments.

### Participants

A total of 9 right handed healthy participants (8 male, 1 female) with a mean age of 27 ± 2 years old participated in the study. Participants were recruited based on their motivation to participate, with no previous known neurological disorder. We included only naïve subjects, with no previous exposure in BCI, to have a first-time user experience (FTUE). This was done in order to minimize any bias by previous experienced in MI in neurofeedback and because our target population has no prior BCI exposure. All participants were students and staff from the University of Madeira and were recruited at the Madeira Interactive Technologies Institute. The experiments were approved by the Ethics Committee of the Public Health System of the Autonomous Region of Madeira, Portugal (SESARAM), with decision number: 15/2015. All subjects were informed and signed an informed consent to participate and to publish their data according to the Declaration of Helsinki.

### Questionnaires

Subjective experience data was gathered through three questionnaires: the *Presence Questionnaire*, the *Vividness of Movement Imagery Questionnaire-2*, and the *NASA TLX*.

- The Presence Questionnaire (PQ) is a tool that measures the degree to which individuals experience presence in a virtual environment and the influence of possible contributing factors to the intensity of the experience [59]. PQ has 24 questions in a seven-point Likert scale to assess items such as realism, possibility to act and sounds. Items related to haptic assessment were excluded because this aspect was not addressed in our experiment.
- Vividness of Movement Imagery Questionnaire-2 (VMIQ2) [60] was used to assess the Kinesthetic Imagery ability of the participant. VMIQ comprises 12 questions to rate vividness of different items in a 5-point scale. Participants had to report how clear was the image obtained by imagining themselves do the following movements (Kinaesthetic imagery): walking, running, kicking a stone, bending to pick up a coin, running up-stairs, jumping sideways, throwing a stone into water, kicking a ball in the air, running downhill, riding a bike, swinging on a rope, and jumping off a high wall. The VMIQ has been previously used to determine differences in neural activation patterns between vivid and non-vivid imagery [61].
- Finally, the NASA TLX questionnaire was used to measure task load through a number of subscales [62]. These subscales include Mental Demands, Physical Demands, Temporal Demands, Performance, Effort and Frustration.

### Data analysis

#### Power Spectral Density (PSD) Estimation

In order to remove major artifacts related with eye blinking and muscular activity, a manual cleaning of the signal in the time domain was performed, followed by a component rejection process. The component rejection was performed by using Independent Component Analysis (ICA) with the help of the EEGLAB toolbox [63]. With the use of ICA we rejected components responsible for major artifacts of either endogenous (muscle, jaw clenching, eye movement) or exogenous source (AC power). EEG rhythms were processed by extracting the Power Spectral Density (PSD) of the signals in Matlab (MathWorks Inc., Massachusetts, US). The power was extracted every 500 ms using Welch’s method with windows of 128 samples for the following frequency bands: Alpha (8 Hz - 12 Hz), Beta (12 Hz - 30 Hz), Theta (4 Hz - 7 Hz), Low Gamma (25 Hz - 45 Hz), and High Gamma (55 Hz - 90 Hz). For the current analysis and because we were only measuring from sensory-motor areas, data were averaged for all the channels for each experimental condition. Moreover, left and right hemisphere electrodes were aggregated to assess hemispheric differences between conditions.

### Statistical analysis

The following metrics are used as dependent variables in our experimental design: EEG rhythm amplitude, MI classifier performance, Workload, and Kinesthetic Imagery.

- EEG Rhythms: We used the *mean* PSD from each EEG frequency band for each condition.
- MI classifier performance: From the LDA classification accuracy on both the calibration and the online task blocks, we calculated the *mean classification accuracy* per condition as a percentage.
- Workload: We used the *sum* of all sub-elements of the TLX questionnaire to extract the Workload for each participant on each condition.
- Kinesthetic Imagery: We used the *sum* of all sub-elements per user to extract the overall Kinesthetic Imagery.

Normality of the distribution of all data was assessed using the Shapiro-Wilk (S-W) normality test, recommended for tests with a sample size of less than 50 [64]. For classifier performance, and because the data deviated from normality, non-parametric statistical tests were used for the analysis. For the assessment of overall differences between the three experimental conditions, a Friedman test was used on each dependent variable. For further pairwise comparisons, the Wilcoxon signed-rank test on each of our combinations was used. On EEG rhythm data, the S-W test revealed normality of the data (*p* > 0.05). We therefore analyzed the data using a repeated measures ANOVA with a Greenhouse-Geisser correction due to Mauchly's Test of Sphericity violation. For all pairwise comparisons a Bonferroni correction was used to account for the number of comparisons. Effect sizes were computed on pairwise comparisons. For all statistical comparisons the significance level was set to 5 % (*p* < 0.05). All statistical analysis was done using IBM SPSS 20 (SPSS Inc., Chicago, IL, USA).

Spearman correlations were performed between the mean PSD from all EEG rhythms (Alpha, Beta, Theta, Gamma) and questionnaire (Workload, Kinesthetic Imagery, and their sub-domains) data, with a significance level set to 5 % (*p* < 0.05).

### Multivariate linear regression

A Stepwise regression modelling approach was used to identify electrophysiological predictors that provide a good fit based on their statistical significance (*p* < 0.05) between subjective (questionnaires) and objective (EEG) data. The set of variables that were used for the multivariate linear regression includes (a) the subjective experience as reported through the questionnaires against (b) the EEG rhythms. The Stepwise coefficient estimation of the models was done using Matlab (MathWorks Inc., Massachusetts, US).

## Results

In the following section, results concerning EEG activity, classification performance and questionnaire answers are illustrated for all conditions. In addition, electrophysiological correlates between subjective and objective data are assessed in order to understand how we can maximally engage motor areas in an MI-BCI task.

### Effect and impact of different MI-BCI experimental paradigms

To assess the difference between all conditions, we compared the different EEG rhythms, the classification score (the ability of the classifier to identify correctly one of the two classes of our motor-imagery task), and the hemispheric asymmetry for (1) motor-execution during MP, (2) VRMP condition, (3) VR condition, and (4) Control condition. In this analysis, (1) and (4) are used both as controls for comparison to standard MI-BCI feedback and to assess resemblance with actual motor-execution. The latter is particularly interesting since we aim for a MI-BCI paradigm that is able to retrain the same motor networks that are responsible for actual movement.

#### Calibration Block

**Fig. 3:**
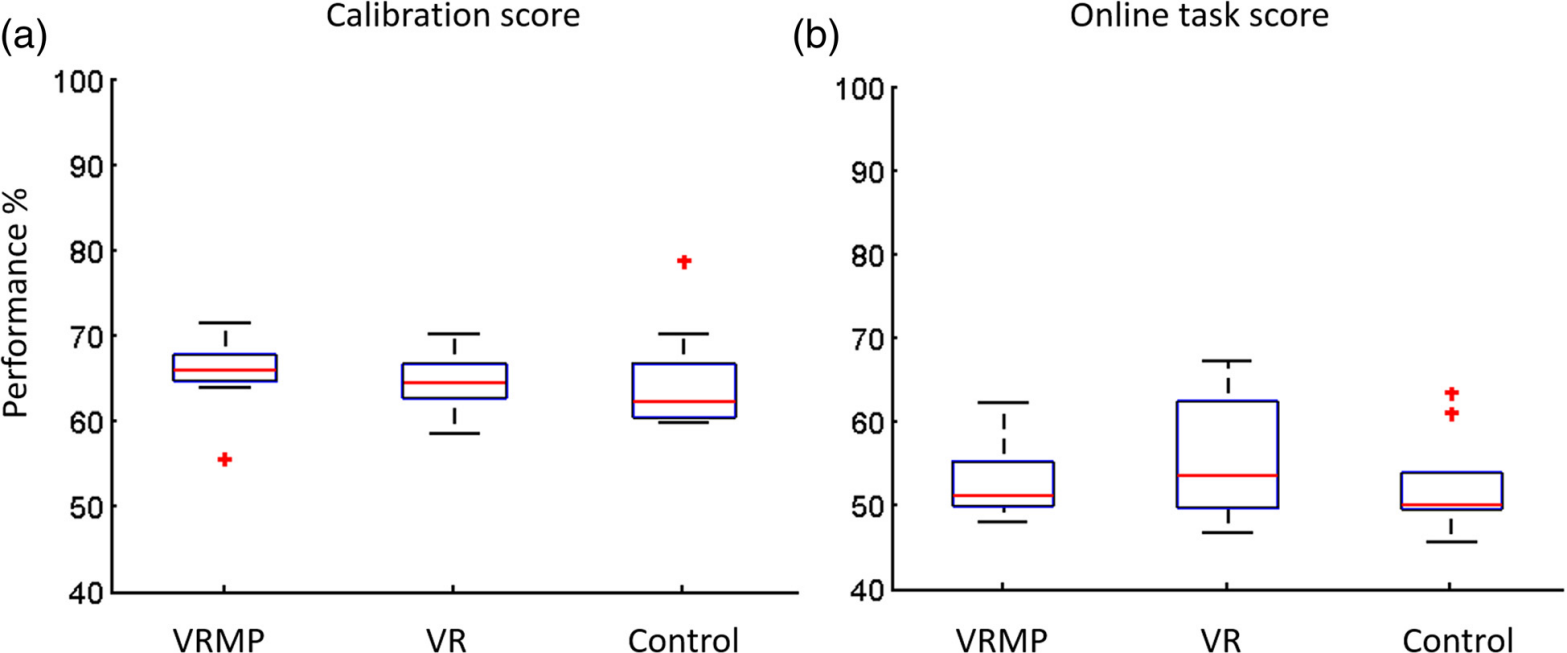
LDA classifier score. (**a**) Calibration score of the LDA classifier illustrating the ability of the classifier to distinguish the left | right imaginative hand movement. (**b**) Online task score, illustrating the ability of the classifier to distinguish the two classes with untrained data
iii. Hemispheric Asymmetry
  In the Calibration block, we observe the same convergence pattern towards motor-execution present in the previous EEG analysis for all frequency bands (Fig. 5a). A repeated measures ANOVA determined that mean difference of hemispheric asymmetry, was not statistically significantly different between conditions for calibration, in Alpha (F(2.219, 17.754) = 0.865, *p* = 0.448), Beta (F(1.905, 15.242) = 0.998, *p* = 0.388), Theta (F(1.941, 15.528) = 0.960, *p* = 0.402), low Gamma (F(2.083, 16.667) = 0.719, *p* = 0.507), and high Gamma (F(2.430, 19.443) = 0.625, *p* = 0.625);

**Fig. 4:**
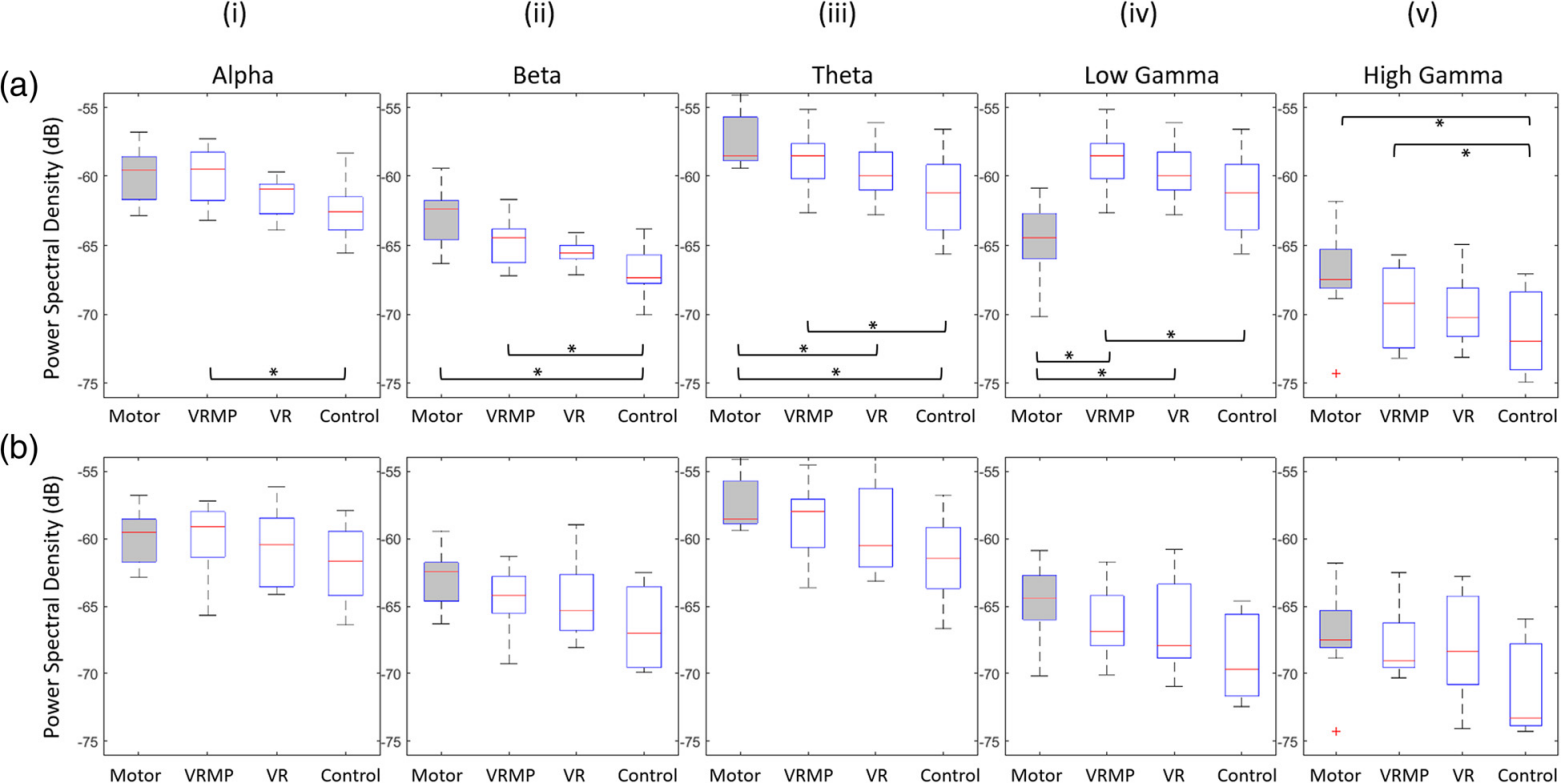
Power Spectral Density (PSD) of all EEG bands. (**a**) EEG band modulation during the calibration session. (**b**) EEG band modulation during the MI task. *p < 0.05
ii. Classification Score
  In contrast to the calibration block, performance score drops considerably (>10 %) for all conditions during the subsequent MI task block, showing lower performances and higher variability (Fig. 3b). Notably, for VRMP, performance dropped to Mdn = 51.29 (*IQR = 6.42*), for VR to Mdn = 53.61 (*IQR = 12.99*) and in Control condition to Mdn = 50.1 (*IQR = 7.23*).
iii. Hemispheric Asymmetry
  A repeated measures ANOVA determined that mean difference of hemispheric asymmetry was not statistically different between conditions for the MI task, Alpha (*F(2.094, 16.754) = 1.210, p = 0.325*), Beta (*F(2.236, 17.891) = 1.519, p = 0.245*), Theta (*F(1.878, 15.023) = 1.263, p = 0.309*), low Gamma (*F(2.299, 18.393) = 1.047, p = 0.380*), and high Gamma (*F(2.287, 18.296) = 1.086, p = 0.366*) (Fig. 5b).

i. EEG rhythms
  A repeated measures ANOVA determined that mean EEG rhythms differed significantly across conditions for: Alpha (*F(2.524, 20.191) = 4.800, p < 0.05*), Beta (*F(1.599, 12.796) = 7.541, p < 0.05*), Theta (*F(1.874, 14.990) = 7.615, p < 0.05*), low Gamma (*F(1.713, 13.701) = 11.639, p < 0.05*), and high Gamma (*F(1.617, 12.938) = 6.869, p < 0.05*) (Fig. 4a). EEG rhythms during calibration show a convergence of brain activation for VR and VRMP conditions towards overt motor-execution. Overall, EEG data show a clear trend with overt motor-execution and Control condition at opposite ends and VR and VRMP in between, being the latter the closest to motor-execution. Post hoc tests revealed that the mean EEG rhythm on the Alpha band differed significantly between VRMP and Control conditions. For the Beta band, a significant difference was found between both motor-execution and VRMP conditions with Control. For the Theta band, motor-execution was significantly different from both VR and Control conditions, and VRMP from Control. In Lower Gamma, motor-execution was significant different from VRMP and VR, as VRMP was significantly different from Control. Interestingly, in Lower Gamma, the above trend was altered, with the mean power of overt motor-execution displaying the lowest values. Finally, for Higher Gamma, there was a significant difference for both motor-execution and VRMP conditions with Control.
ii. Classification Score
  The MI-BCI calibration data revealed that the multimodal setup with motor priming condition (VRMP) provided the highest performance (*Mdn = 65.8, IQR = 3.32*) when compared with the VR only condition (*Mdn = 64.5, IQR = 5.41*) and control condition with the traditional feedback (*Mdn = 62.3, IQR = 7.63*) (Fig. 3a). However, these differences are small and a Friedman test revealed no statistical difference (*χ2(2) = 1.429, p = 0.490*).

#### MI Task Block

i. EEG Rhythms
  The mean EEG rhythms during the MI task block followed a very similar trend as in the calibration block (Fig. 4b), being both blocks significantly correlated for Alpha (*r = 0.564, p < 0.01*), Beta (*r = 0.501, p < 0.01*), Theta (*r = 0.599, p < 0.01*), low Gamma (*r = 0.555, p < 0.01*), high Gamma (*r = 0.635, p < 0.01*). The repeated measures ANOVA revealed a significant difference for Theta (*F(2.660, 21.277) = 3.520, p < 0.05*). Nevertheless, no statistical differences across conditions were found for Alpha (*F(2.804, 22.429) = 0.813, p = 0.493*), Beta (*F(2.628, 21.020) = 2.780, p = 0.72*), low Gamma (*F(2.434, 19.475) = 3.199, p = 0.055*), and high Gamma (*F(2.232, 17.860) = 3.071, p = 0.067*). Post hoc tests using the Bonferroni correction revealed that there is a trend for VRMP against the control condition (*p = 0.073*) but not for the rest of the pairwise comparisons. Interestingly, the mean power of the Lower Gamma frequency band was reduced for all MI conditions, showing that EEG activation during the MI task block was more similar to motor-execution than in the calibration block, and hence in accordance with the trend identified in the rest of frequency bands (Fig. 4).

**Fig. 5:**
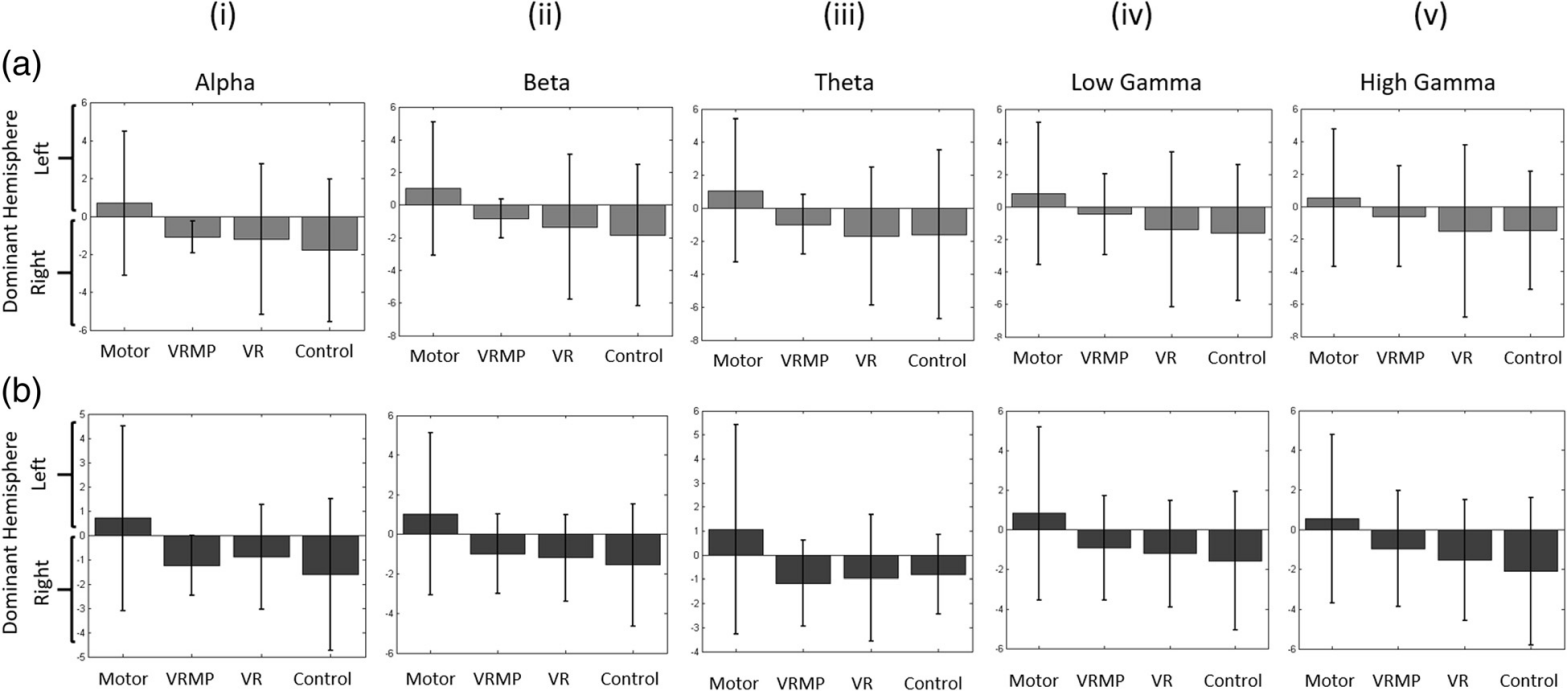
Hemispheric Differences between Left and Right EEG activation. (**a**) Hemispheric differences of the EEG rhythms during calibration. (**b**) Hemispheric differences of the EEG bands during MI task performance

### Quality of the experience

In order to understand how different MI training paradigms may affect the quality of the experience and the overall acceptance of the system, we analyzed a set of subjective data as reported by the participants, including the sense of Presence, Kinesthetic Imagery ability, and perceived Workload for each condition.

**Fig. 6:**
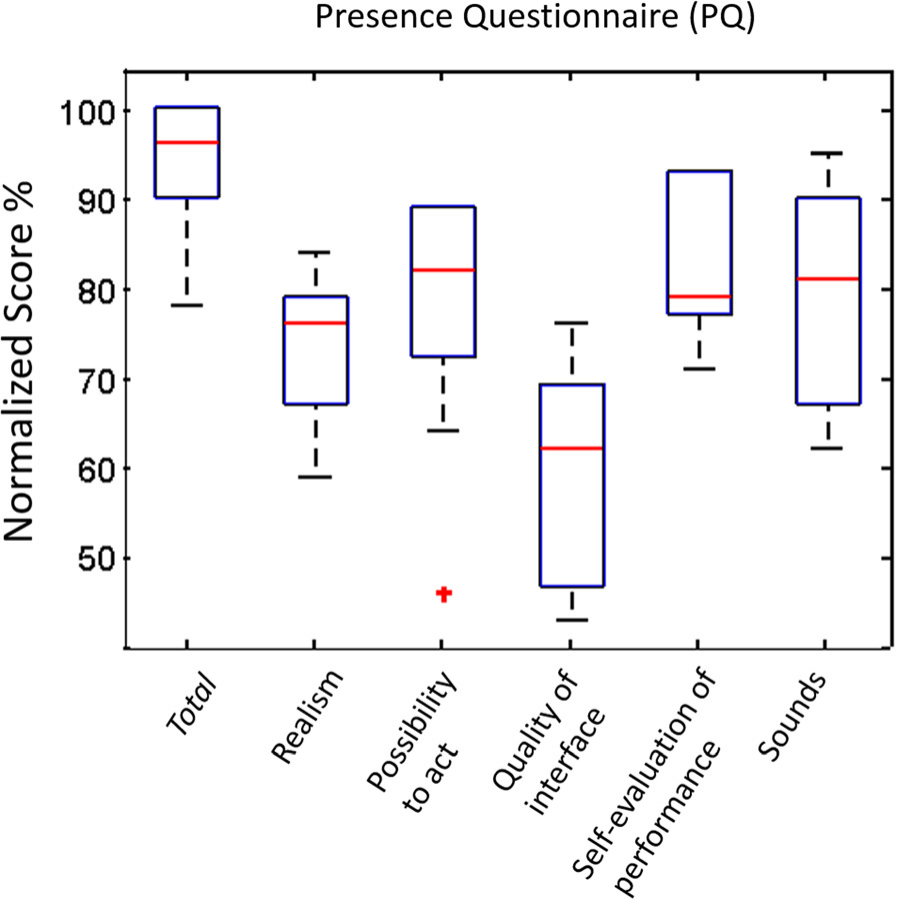
Presence Questionnaire normalized total score (gray) and the sub-domains. Four out of the five domains scored above 70 %, with quality of the interface to score the lowest
- Correlates of Workload, Kinesthetic Imagery and Task Engagement
  After the MI task block on each condition, the perceived Workload was assessed through the NASA TLX questionnaire and the Kinesthetic Imagery ability through the VMIQ-2 questionnaire. A repeated measures ANOVA determined that mean Workload differed significantly across conditions (*F(1.505, 12.036) = 5.290, P < 0.05*) (Fig. 7). Post hoc tests revealed that Workload in the VRMP condition to be significantly higher than for Control. A correlation analysis revealed no correlation between Workload and the performance during the MI task block.

- Realism of the VR Training Simulation
  Both VRMP and VR conditions share the same virtual environment for which users were asked to report their sense of presence. The normalized score of the Presence Questionnaire (PQ) indicates an overall acceptance of the VR task (*M = 94.3 %, SD = 8.3)* (Fig. 6). Overall, four out of the five domains considered scored above 70 %: realism (*M = 73 %, SD = 8*), the possibility to act through initiated actions and events (*M = 77 %, SD = 14*), sounds of the VR task (*M = 79 %, SD = 12*), and the self-evaluation of performance, which had the highest score (*M = 83 %, SD = 9*). The quality of the interface showed the lowest score (*M* = 58 %, *SD* = 13). Nevertheless, the quality of the interface did not seem to affect the high perceived performance and realism of the VR task.

**Fig. 7:**
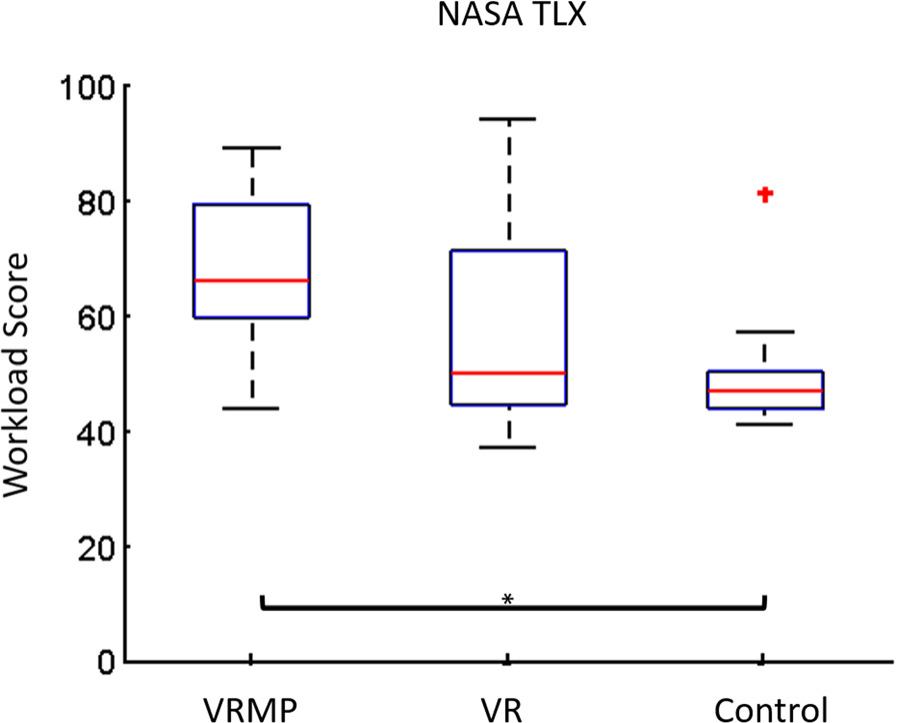
NASA TLX questionnaire for perceived Workload. VRMP condition is the most demanding in terms of task workload. *p < 0.05
  Kinesthetic Imagery was assessed through the VMIQ-2 questionnaire. The cut-off-point established by Whetstone estimates good imagery ability with a total score of 70 % [65]. Our experiment considered only first-time user experiences, and the average ability score was *61.36 % (SD = 12)* and only 3 out of 9 subjects scored above 70 %. A comparison among conditions showed that conditions did not affect the participant’s ability to create clear and vivid motor imagery (*F(1.567, 12.532) = 1.292, p = 0.300*) (Fig. 8). A correlation analysis showed no significant correlation between Kinesthetic Imagery and the performance during the MI task block.

**Fig. 8:**
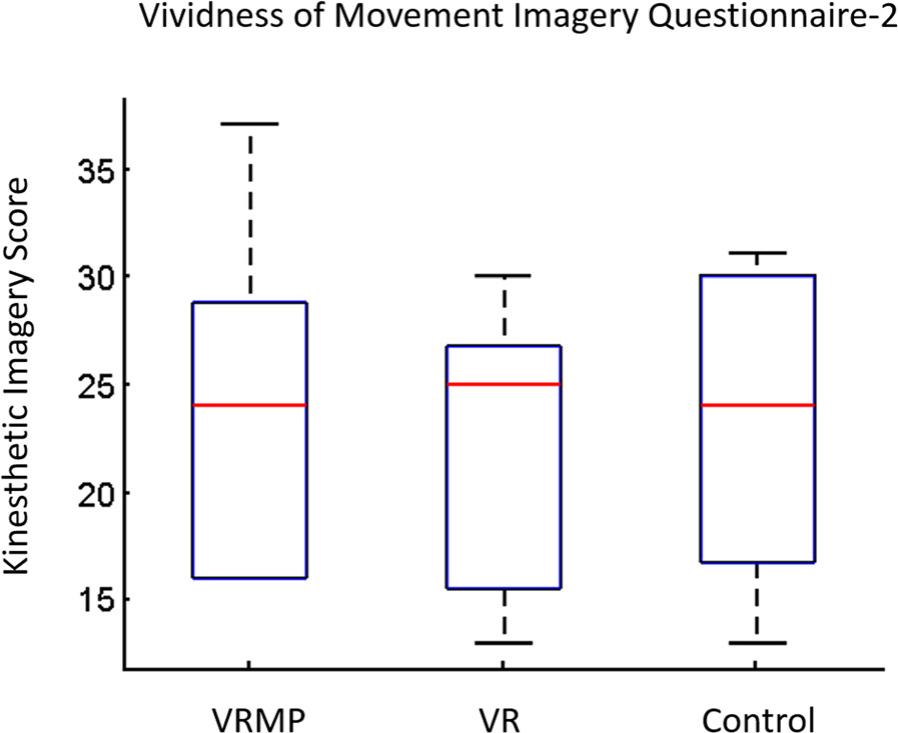
Kinesthetic Imagery (KI) score through the Vividness of Movement Imagery Questionnaire-2 (VMIQ2). Through all conditions, users had a consistent Kinesthetic Imagery ability and was not got affected across conditions

### Relationship between EEG rhythms and subjective experience

In order to identify which patient profile can benefit the most from an immersive BCI-VR setup, we investigated the relationship between subjective experience (as reported through the TLX and Kinesthetic Imagery questionnaires) and the elicited brain activity patterns (Alpha, Beta, Theta, and Gamma EEG rhythms; and the EI). The following section illustrates the findings that have been extracted through correlation and multilinear regression modelling analyses.

- Correlation Analysis
  Considering only the EEG data during the MI task block, we identified correlations of Alpha and Theta bands with the subjective reports (Table 1)*.* For the TLX subcomponent of Mental Demand we found a significant correlations with Alpha (*r = 0.500, p < 0.05*) and Theta (*r = 0.555, p < 0.05*). Negative correlations were found for Alpha with the reported Kinesthetic Imagery ability in Jumping Sideways (*r = −0.381, p < 0.05*) and Running Downhill (*r = −0.420, p < 0.05*), and for Theta only for Running Downhill Kinesthetic Imagery (*r = −0.545, p < 0.05*).

**Table 1:** Correlation table from MI task EEG data including Alpha and Theta bands with TLX and its subdomains

	TLX - Mental Demand	KI - Jump Sideways	KI – Run Downhill
Alpha	0.500	−0.381	−0.420
Theta	0.555	–	−0.545
- Multilinear Regression Modelling
  A stepwise regression modelling was used to identify electrophysiological predictors of subjective experience based on EEG PSD and questionnaire data (Table 2). Mental Demand was found to relate to a combination of Theta and Beta bands (*F(2, 24) = 8.894, p < 0.05, R*^*2*^ *= 0.426*). Interestingly, although both Alpha and Theta bands were shown to positively correlate with Mental Demand, this is better explained through Beta and Theta. This may indicate collinearity between Alpha and Theta bands. For Kinesthetic Imagery, Alpha band modulation is related to the user’s capacity for mental imagery that involves sideways jumps (*F(1, 25) = 4.607, p < 0.05, R*^*2*^ *= 0.156*), and Beta and Theta for mental imagery that involves running downhill (*F(2, 24) = 10.606, p < 0.05, R*^*2*^ *= 0.469*).

**Table 2:** Stepwise model coefficients from online data

	TLX – Mental Demand	KI – Jump sideways	KI – Run downhill
x_1_ : Alpha	–	- 0.123	–
*x*_2_ : Beta	1.638	–	0.204
x_3_ : Theta	−1.107	–	−0.273
R^2^	0.426	0.156	0.469

Electrophysiological predictors of Alpha, Beta, and Theta, based on their statistical significance. (*p < 0.05*) between the questionnaires and their sub-domains

## Discussion

The obtained results contribute with a set of important findings in several dimensions: quantification of EEG modulation and classification through VR feedback and MP, and how those relate to perceived experience and Kinesthetic Imagery ability. These findings may be important to enhance the impact of MI-BCI in neurorehabilitation and push the state-of-the-art.

Firstly, through the analysis of EEG rhythms we compared VR and VRMP conditions with (1) a standard control condition using Graz visualization and (2) actual EEG activity during overt motor-execution. Our EEG data revealed statistically significant differences of VRMP with standard feedback, suggesting the engagement of different underlying processes, more consistent with motor-execution data. The differences in Alpha and Beta with control and their similarity with the activity induced during motor-execution is of high importance for MI training in rehabilitation due to better association to cortical activation of sensorimotor areas during voluntary movement [66, 67]. Furthermore, increased activity in Alpha and Theta could indicate an effect of increased cognitive and memory load in VR [6], as also shown in our study through TLX data. However, despite measurable differences in EEG activity among conditions, these did not significantly change the classification performance of the LDA used for BCI control.

We also observed in our hemispheric asymmetry analysis that interhemispheric communication changed during the different MI-BCI paradigms. Previous studies have shown that the hemispheric asymmetry increases during feedback presentation compared to sessions without feedback [49], enhances the performance of fine motor tasks and triggers changes in motor learning [50]. A recent study highlights that the left hemisphere is specialized for sequential motor organization in both left- and right-handers, suggesting an endogenous hemispheric asymmetry related to compound actions and skill representation [68]. Therefore, if interhemispheric communication can be modulated through VRMP as our data suggests, this is an important feature to be utilized in motor learning. In patient populations with affected hemispheric differences we could promote increased interhemispheric interaction by balancing the activation of motor-areas and influence motor performance [69]. In addition, interhemispheric interactions may also contribute to intermanual transfer, as it has been found that motor learning using one hand improves the performance of the other hand [70, 71]. Therefore, longitudinal neuroimaging and electrophysiological studies are necessary in order to demonstrate the dynamic change in interhemispheric interaction between both hemispheres during the process of functional recovery in stroke survivors.

Secondly, subjective data reported through questionnaires allowed us to report on their relationship with EEG data, providing insights of the effect of different MI conditions in both of cognitive and motor processes. Interestingly, although in the VRMP condition the user had to exert more physical activity, our data revealed that Physical Demand and Effort subcomponents of the TLX were not affected. We argue that the inclusion of the MP component within an immersive VR environment turned the MI-BCI task into a more mentally demanding task, with the potential of engaging more neural circuits than in the other 2 conditions. This hypothesis is also supported by the differences found in the EEG activity patterns. Additionally, we found a correlation between Kinesthetic Imagery ability and their capacity to display enhanced activity in the Alpha and Beta bands, which are modulated during cortical activation/deactivation in the planning of voluntary movement [72, 73]. Finally, enhanced sensory-motor rhythms through MI-BCI training have been shown in patients displaying higher motor improvements as assessed by the Fugl-Mayer [74]. Thus, our findings give further support to the importance of the vividness of motor-imagery capability in MI-BCI training, −especially the walking components of the questionnaire (jump, run)-, enabling us to use them as inclusion criteria in a neurorehabilitation MI-BCI paradigm, considering that their reliability has been assessed in both healthy and post-stroke people [75].

## Conclusions

Our findings are aligned with previous research, verifying that abstract feedback versus realistic, can have very little effect in terms of BCI classification performance, but showing that BCI feedback clearly modulates sensorimotor EEG rhythms [15]. This could lead towards better functional outcomes compared with standard MI as reported by previous research [74].

Our current results are based on the premise that it is possible to modify EEG rhythms through multimodal feedback, affecting the activity of somatosensory and motor areas for the better. This is a proposition for which there is limited empirical evidence so far. We found consistent performance trends related to the type of interface but also enhanced EEG rhythms modulation through immersive VR and motor priming. Overall, we showed that, both VR conditions elicited an increase of mean power in all EEG rhythms. Although it is known that motor-imagery involves to a large extent the same cortical areas that are activated during actual motor preparation and execution [66], we have shown that motor-imagery training in a multimodal setup and priming (VRMP) can provide the strongest and most similar motor network activation to overt movement-execution from all tested MI-BCI training paradigms. Furthermore, the activation of ipsilateral (contralesional) primary sensorimotor cortex (SMC) and the mirror neuron system (MNS) appears to play a fundamental role in both action execution and imitation [67, 76, 77] enhanced by VR. With current findings in hemispheric asymmetry, we can distinguish the important role of interhemispheric communication in motor learning.

Moreover, by assessing the quality of the experience, we observed a high overall acceptance of the novel multimodal MI paradigms, despite a reported increase in Workload. By modeling electrophysiological data and perceived experience data, we are able to better describe the relationship between user profile (Kinesthetic Imagery ability, perceived Workload, Presence in VR) and EEG rhythms changes in response to MI-BCI training, which may become very relevant to identify which patients can benefit the most from it.

In practice, satisfactory BCI control depends largely on the degree to which neural activity can be voluntarily controlled by users. Therefore, approaches to the training of users to control a BCI taking into consideration the specific target population play an important role. In the case of stroke survivors, our approach is based on the priming of the sensorimotor system, through realistic VR and training through gamified tasks. For patients with severe hand paresis for who motor priming through movements of the paretic limb is not possible, a VR setup such as ours could offer the ability to mirror the healthy arm during the priming session, with the affected. Mirror therapy is the use of visual illusion created by a mirror by superimposing the intact arm over the paretic. Mirror therapy is well established in stroke rehabilitation for promoting recovery [78, 79]. Therefore, our system could also be used to provide MI driven mirror therapy by mirroring the healthy arm to virtual limbs. Overall, in this study we showed that MI training with multimodal setup and priming (VRMP) is an effective paradigm to elicit sensorimotor activation consistent with motor execution. We showed that thanks to our quantification of the perceived experience in MI-VR training could improve adherence to the treatment by adjusting the VR task to improve the experience. Finally, the proposed VRMP paradigm has a large potential even in the case of patients with no motor control, by exploring the possibilities of MI-BCI driven mirror therapy.

In the future, we plan to run a study with stroke participants in order to evaluate the impact of the proposed VRMP paradigm in motor function restoration. We plan to clinically validate the VRMP paradigm in a longitudinal 1-month MI-BCI study including motor evaluations and pre- and post- functional brain imaging to identify the underlying neural activation and reorganization correlates of motor recovery.

### Study limitations

This study, although it results from the collection of 63 EEG datasets, is limited by its sample size (*N* = 9). Findings have limited statistical power and should be interpreted with caution. Moreover, results from healthy participants cannot be directly generalized to a stroke population, which requires further research. Finally, current data was recorded through 8 EEG electrodes and a limited spatial resolution. An increased amount of electrodes could offer new insights by covering a larger area of the sensorimotor and neighboring areas and with higher resolution.

## Abbreviations

BCI, brain-computer interface; EEG, electroencephalography; MI, motor-imagery; SMR, sensorimotor rhythm; VR, virtual reality; VRMP, Virtual Reality with Motor Priming
